# Shotgun proteomic analysis of *Anaeroglobus geminatus*

**DOI:** 10.1080/20002297.2017.1325252

**Published:** 2017-05-30

**Authors:** Kai Bao, Nagihan Bostanci, Thomas Thurnheer, Georgios N. Belibasakis

**Affiliations:** ^a^ :Division of Oral Microbiology and Immunology, Institute for Oral Biology, Center of Dental Medicine, University of Zürich, Zürich, Switzerland; ^b^ Department of Dental Medicine, Karolinska Institute, Stockholm, Sweden

## Abstract

**Objectives**: *Anaeroglobus geminatus* is a gram-negative species belonging to the family of *Veillonellaceae* and close associate to genus *Megasphaera*. The role of the species within dysbiotic microbial communities is not yet clear. However, this putative pathogen has been identified in a number of anaerobic infections, including periodontal diseases. The aim of this proteomic study was to analyse the global protein expression of *A. geminatus*.

**Methods**: The whole proteome of *A. geminatus* CCUG 44773 strain was analysed by shotgun proteomics, using Orbitrap Fusion mass spectrometer.

**Results**: A total of 1164 *A. geminatus* proteins were identified by this analysis, with an FDR of 2.2 % and 0.19 %, at peptide and protein level, respectively. Functional analysis of the identified proteins was performed using the Uniprot knowledge-base. Among those, 100 proteins were predicated to be transferase, 47 were hydrolases, and 41 were ligases, based on computational analysed functions. The top-scored pathways were “amino-acid biosynthesis” and “cofactor biosynthesis”, and each of these pathways included 20 proteins, such as arginine repressor and aspartokinase.

**Conclusion**: According to our knowledge, this is the first study that gives an overview of the global proteome of *A. geminatus* and hopefully will help to unravel its role in anaerobic infections.

